# Biodegradable Plastic Mulch Films: Impacts on Soil Microbial Communities and Ecosystem Functions

**DOI:** 10.3389/fmicb.2018.00819

**Published:** 2018-04-26

**Authors:** Sreejata Bandopadhyay, Lluis Martin-Closas, Ana M. Pelacho, Jennifer M. DeBruyn

**Affiliations:** ^1^Department of Biosystems Engineering & Soil Science, The University of Tennessee, Knoxville, Knoxville, TN, United States; ^2^Department of Horticulture, Botany and Gardening, School of Agrifood and Forestry Science and Engineering, University of Lleida, Lleida, Spain

**Keywords:** biodegradable plastic, plastic mulch, polyethylene, specialty crops, soil microbiology, soil microclimate, soil biogeochemistry, soil health

## Abstract

Agricultural plastic mulch films are widely used in specialty crop production systems because of their agronomic benefits. Biodegradable plastic mulches (BDMs) offer an environmentally sustainable alternative to conventional polyethylene (PE) mulch. Unlike PE films, which need to be removed after use, BDMs are tilled into soil where they are expected to biodegrade. However, there remains considerable uncertainty about long-term impacts of BDM incorporation on soil ecosystems. BDMs potentially influence soil microbial communities in two ways: first, as a surface barrier prior to soil incorporation, indirectly affecting soil microclimate and atmosphere (similar to PE films) and second, after soil incorporation, as a direct input of physical fragments, which add carbon, microorganisms, additives, and adherent chemicals. This review summarizes the current literature on impacts of plastic mulches on soil biological and biogeochemical processes, with a special emphasis on BDMs. The combined findings indicated that when used as a surface barrier, plastic mulches altered soil microbial community composition and functioning via microclimate modification, though the nature of these alterations varied between studies. In addition, BDM incorporation into soil can result in enhanced microbial activity and enrichment of fungal taxa. This suggests that despite the fact that total carbon input from BDMs is minuscule, a stimulatory effect on microbial activity may ultimately affect soil organic matter dynamics. To address the current knowledge gaps, long term studies and a better understanding of impacts of BDMs on nutrient biogeochemistry are needed. These are critical to evaluating BDMs as they relate to soil health and agroecosystem sustainability.

## Introduction: Agricultural Plastic Mulch Films

Agricultural plastic mulch films are used in production of specialty crops to modify soil temperatures, conserve soil moisture (Kader et al., 2017) and reduce weed pressure (Martín-Closas et al., 2017), ultimately improving crop productivity. Low-density polyethylene (PE) is the most commonly used plastic mulch because it is inexpensive, easily processed, highly durable and flexible (Kasirajan and Ngouajio, 2012). However, widespread use of PE, which is not biodegradable, has resulted in serious environmental contamination (Teuten et al., 2009; Liu E.K. et al., 2014; He et al., 2015; Steinmetz et al., 2016).

A growing concern is that plastic mulches are never completely removed from a field, leaving remnants which remain in soil for decades (Feuilloley et al., 2005; Kyrikou and Briassoulis, 2007; Briassoulis et al., 2015; Ramos et al., 2015). In China, long term use of plastic film mulches has resulted in an estimated accumulation of 50–260 kg hm^-2^ of residual plastics in topsoil (0–20 cm), which can inhibit plant growth (Liu E.K. et al., 2014). While PE is considered to be chemically inert, accumulated PE fragments can affect soil physically and may enter the food chain (Barnes et al., 2009; Teuten et al., 2009; Sivan, 2011; Rillig, 2012; Duis and Coors, 2016; Huerta Lwanga et al., 2016). Plastic mulches also introduce various additives such as plasticizing agents which may pollute soil (Van Wezel et al., 2000; Fu and Du, 2011; Kong et al., 2012; Magdouli et al., 2013; Wang et al., 2013, 2015).

Biodegradable plastic mulches (BDMs) have been developed as substitutes to PE mulch films and are designed to be tilled into soil after use where resident microorganisms degrade the plastic. BDMs can be prepared from biobased polymers derived from microbes or plants, or fossil-sourced materials (Marechal, 2003). Common biobased polymers used in BDMs include polylactic acid (PLA), starch, cellulose, and polyhydroxyalkanoates (PHA). Fossil-sourced polyesters used in BDMs include poly(butylene succinate) (PBS), poly(butylene succinate-co-adipate) (PBSA), and poly(butylene-adipate-co-terephthalate) (PBAT) (Kasirajan and Ngouajio, 2012). Polymers used in BDMs contain ester bonds or are polysaccharides, which are amenable to microbial hydrolysis (Brodhagen et al., 2015). In theory, BDMs should be completely catabolized by soil microorganisms, converted to microbial biomass, CO_2_ and water (Malinconico et al., 2002; Feuilloley et al., 2005; Imam et al., 2005; Dintcheva and La Mantia, 2007; Kyrikou and Briassoulis, 2007; Kijchavengkul et al., 2008; Lucas et al., 2008). In practice, complete breakdown in a reasonable amount of time is not always observed (Li et al., 2014b). Regulators and growers cite concerns about unpredictable or incomplete breakdown and the ultimate fate of BDM constituents and their effect on soil ecosystems (Goldberger et al., 2015; Miles et al., 2017). Due to increased demand for eco-friendly substitutes to PE, the global market for BDMs is expected to continue to grow. Soil health is a key component of agroecosystem sustainability, thus there is a need to understand the effects of BDMs on both crop productivity and soils. To date, the majority of soil studies related to plastic mulching have focused on PE. The objective of this review is to highlight research concerning impacts of plastic mulches on soil microbial communities and their processes with an emphasis on BDMs. Gaps in our current understanding of how plastics affect soil ecosystems are highlighted.

## Indirect Effects of Plastic Mulches on Soils via Microclimate Modification

**FIGURE 1:**
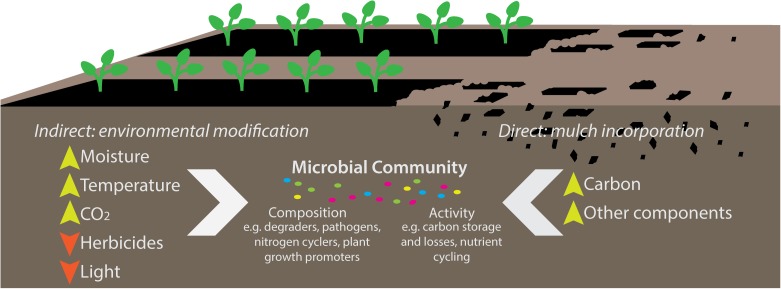
Indirect [polyethylene and biodegradable mulches (BDMs)] and direct (BDMs only) effects of plastic mulching on soil ecosystems. Plastic mulches form a barrier on the soil surface which influences soil temperature, moisture and soil-air gas exchange, indirectly altering the microbial communities. BDMs are tilled into the soil at the end of the growing season, introducing physical fragments and a carbon source, along with other components of the plastic films (additives, plasticizers, minerals, etc.) which may additionally influence soil communities and their processes.

One way that plastic mulches (both BDMs and PE) may indirectly affect soil ecosystems and microbial community functioning is via modification of soil microclimate and atmosphere. As a barrier on the soil surface, plastic mulches reduce evaporation and gas exchange, increase temperature and reduce light transmissivity (**Figure 1**; Kasirajan and Ngouajio, 2012). The extent of these modifications depends on their physicochemical properties; for example, PE mulches result in greater warming compared to BDMs (Moreno and Moreno, 2008; Kader et al., 2017) and are less vapor-permeable (Touchaleaume et al., 2016) resulting in accumulation of soil CO_2_ (Zhang et al., 2015; Yu et al., 2016). By serving as a barrier to evaporation, plastic mulches can result in increased soil moisture levels (Qin et al., 2015) which can ultimately alter soil physical structure; for example by increasing the proportion of water stable aggregates (Siwek et al., 2015). Favorable moisture and temperature conditions under plastic mulches also affect plant roots, typically stimulating root development and increasing root exudation (Li et al., 2004b; Subrahmaniyan et al., 2006; Wang et al., 2016). This results in greater nutrient availability for rhizosphere microorganisms (Subrahmaniyan et al., 2006; Lin et al., 2008; Maul et al., 2014; Liu et al., 2015).

Since levels of soil moisture, temperature, vapor diffusivity and presence of roots modulate microbial activity, it follows that modifications to soil microclimate under plastic mulches affect soil microbial communities. Plastic mulching can also decrease populations of soil invertebrates (Schonbeck and Evanylo, 1998; Miñarro and Dapena, 2003), which may reduce top-down grazing pressures on soil microbes. A 28-year study in Shenyang, China, demonstrated that plastic film mulching increased relative abundances of *Proteobacteria* and *Actinobacteria* (Farmer et al., 2017). Other studies reported improved control of *Phytophthora capsici* (Núñez-Zofío et al., 2011) or increased mycotoxigenic fungi under plastic mulches (Munoz et al., 2015). From PE studies, we can infer that BDMs may have similar indirect effects and alter microbial community structure and diversity.

In addition to changes in microbial community structure, plastic film mulches affect microbial functioning. Some studies report increased microbial activity under mulches (Mu et al., 2014, 2016; Zhang et al., 2015; Chen H. et al., 2017), while others report decreased activity (Moreno and Moreno, 2008). The response is most likely dependent on the amount of warming under the mulches: where ambient temperatures are cool, mulches bring soil temperature closer to microbial optima and increase activity, whereas in warmer seasons, the mulches may push temperatures above optima, limiting soil microbial activity (Moreno and Moreno, 2008). The changes in microbial activity ultimately influence nutrient cycling and storage. The effect of plastic on soil organic carbon (SOC) is the result of the balance between increased root growth and exudate secretion, and microbial decomposition and loss to CO_2_ (Wien et al., 1993; Nan et al., 2016). Thus, it is not surprising that studies examining SOC under plastic mulches have yielded mixed results, with some reporting increased microbial biomass carbon (Li et al., 2004a; An et al., 2015) and SOC (Munoz et al., 2017) and others no change (Wang et al., 2016) or decreased SOC (Cuello et al., 2015). It should be noted that changes in SOC take place over longer time intervals, so the short term (one or two seasons) nature of most mulching experiments do not capture these longer term dynamics. Plastic mulching also affects cycling and losses of nitrogen in soils (Qin et al., 2015; Nan et al., 2016). Because plastic mulching improves water use efficiency (WUE), nitrate leaching is reduced (Romic et al., 2003). Indeed, Qin et al. (2015) estimated up to 60% increase in nitrogen use efficiency (NUE) under PE mulching compared to no-mulch controls. With respect to N_2_O gas release, results are mixed, with some studies reporting decreases (Berger et al., 2013; Li et al., 2014d; Liu J.L. et al., 2014) and others, increases (Okuda et al., 2007; Arriaga et al., 2011; Nishimura et al., 2012; Cuello et al., 2015; Chen H. et al., 2017).

Together, these studies show that plastic mulching, independent of composition, has significant effects on soil microbes and their processes via environmental modification. In several cases, improved crop productivity with mulch was accompanied by a loss of soil organic matter and increased release of greenhouse gasses (Steinmetz et al., 2016). It is important to note that PE films often result in higher soil temperatures and are more effective in suppressing weeds compared to BDMs (Bonanomi et al., 2008). As a physical barrier, BDMs are expected to have similar, though not identical, indirect effects on soil microbes via microclimate modification; the outstanding question is how these effects play out when direct incorporation and biodegradation of BDMs are taken into consideration.

## Direct Effects of BDMs via Incorporation Into Soil

While BDMs may have comparable effects as PE mulches when used as a surface barrier, they are distinctly different when considering their ultimate fate. After the growing season, PE films should be removed from the soil surface, while BDMs are meant to be tilled in and biodegraded by microorganisms. BDM fragments are both a physical and a biogeochemical input (**Figure 1**). This aspect is unique to BDMs, and may have effects on soil ecology and functioning that cannot be predicted from studies of non-biodegradable plastics such as PE.

Biodegradable plastic mulch fragments may physically modify soil before they are fully biodegraded. For example, PE plastic fragments reduce soil infiltration and water absorption; their accumulation may affect soil ecosystems and ultimately plant germination and growth (Liu E.K. et al., 2014). Therefore, it is conceivable that under conditions restricting soil microbiological activity (e.g., water scarcity), BDM fragments may accumulate in soil with similar effects on soil and plants.

From a toxicology standpoint, the fragments of BDMs incorporated into the soil are generally considered to be safe. For example, tests of the starch-copolyester blend Mater-Bi^®^ (Novamont, Novara, Italy) have shown no ecotoxic effects (Sforzini et al., 2016), nor adverse effect on nitrification potential (ISO 14238:2012) (Ardisson et al., 2014), *Enchytraeus albidus* reproduction *(*ISO/CD 16387), or *Vibrio fischeri* (ISO 11348 flash test) (Kapanen et al., 2008). Similarly, soil samples containing Ecoflex^®^ (BASF), PHB, and PLA show no demonstrated visual phytotoxicity (ISO 11269-2) (Rychter et al., 2006, 2010). It should be noted that these studies focus on acute responses; possible effects of longer exposure is untested.

Plastic mulches are composed not only of the main polymers but also of small amounts of organic (e.g., additives, plasticizers, etc.) and inorganic (e.g., Cu, Ni, etc.) components, whose effects are largely unknown. Traditional plant tests for toxicity have not been adapted to identify effects of compounds released from BDMs. First, different compounds are released at different times during the biodegradation process. Second, frequently used tests fail to reckon the changing needs and responses throughout plant development by only focusing on germination. Finally, the diversity of plant responses in the ecosystem is narrowly represented by tests that analyze early growth in a few, mostly vigorous, plant species. Despite these constraints, some effects have emerged. A phytotoxicity test of several chemicals used in bioplastics found that some exhibited a concentration-dependent inhibition of plant growth (Martin-Closas et al., 2014). Acrylate polymers used to maintain soil humidity damaged maize root and shoot development (Chen et al., 2016). Organic compounds released from mulch polymers have been found to be absorbed by crop plants (Du et al., 2009; Li et al., 2014c; Chen N. et al., 2017). Given some of the demonstrated effects on plants, these additives may also impact soil microbes and their functions, though these effects are largely unexplored.

Tilled into soil, BDMs are an input of carbon, albeit a very small one when taking into account the volume of soil into which they are incorporated. However, the growth of soil microbes in agricultural soil is usually carbon-limited and several studies have demonstrated responses by soil microbes to these small inputs. BDMs have caused increases in microbial biomass and enzyme activities (Li et al., 2014a; Yamamoto-Tamura et al., 2015) and changes in soil microbial community structures (Koitabashi et al., 2012; Li et al., 2014b; Muroi et al., 2016). There is evidence that BDMs enrich for certain taxa, for example, PBSA films preferentially selected for *Aspergillus, Penicillium*, and *Acanthamoeba* fungi (Koitabashi et al., 2012) and PBAT film surfaces were enriched in Ascomycota *(Apodus, Saccharicola, Setophoma)*, and Proteobacteria *(Hyphomicrobium, Caenimonas*) (Muroi et al., 2016). Several studies have also noted increased fungal abundances in soil as a result of BDM incorporation (Rychter et al., 2006; Li et al., 2014b; Ma et al., 2016; Muroi et al., 2016). The majority of these studies examine only one soil type or location; one of the few studies to examine responses in multiple locations showed an enrichment of fungi in one location and Gram-positive bacteria in another (Li et al., 2014b) indicating that microbial responses to BDMs may be affected by environment, soil type and/or management legacies.

In order to tease apart whether observed changes in microbial communities are a result of microclimate effects (i.e., changes that would be expected regardless of the plastic material used) or are specific to BDMs tilled into soil, results from studies that directly compare microbial communities under PE and BDMs in the same experiment are required. The few studies available reported increased microbial abundances, respiration, and enzyme activities under BDMs compared to PE treatments (Moreno and Moreno, 2008; Li et al., 2014a; Yamamoto-Tamura et al., 2015; Barragán et al., 2016; Hajighasemi et al., 2016; Ma et al., 2016) suggesting that incorporation of BDMs does have some effect on microbial activity. Evidence of enhanced degradative activities by soil microbes suggests that BDMs may ultimately change carbon cycling and storage in soil. The total amount of carbon in BDMs is small, and much of it is expected to be respired as CO_2_. However, repeated tilling of BDMs into soil may have an effect over time. In one study, use of BDMs resulted in increased microbial biomass carbon compared to PE mulches (Moreno and Moreno, 2008), suggesting an impact on soil carbon dynamics that may accumulate over time. It should also be considered whether enhanced BDM decomposition would impact cycling of other nutrients. Studies on nutrient transformation related to BDM use are limited; two studies reported that BDMs, like PE films, had no measurable impact on nitrification potential of soils (Kapanen et al., 2008; Ardisson et al., 2014); effects on other nutrients remain unknown.

Taken together, the changes in microbial community structures, stimulated microbial decomposition, and increased microbial biomass suggest enhanced nutrient and carbon cycling under BDMs, which may result in long term effects on soil organic matter dynamics. However, with limited research on long term studies, it remains unknown if BDMs may impact soil functions differently than PE and what implications this has for sustainability of this technology for crop production.

## Future Research Opportunities

Biodegradable plastic mulches are a promising alternative to PE plastic film mulches. However, there are considerable gaps in our understanding of how long-term use of BDMs affects soil ecosystems that are critical to crop productivity. Effects of conventional PE mulches on soil microclimate, microbial communities and biogeochemistry provide insight into how BDMs may be indirectly influencing soil. As a surface barrier, plastic mulches can alter soil microbial community composition and functioning in terms of carbon and nitrogen cycling via microclimate modification, though the nature of these alterations has varied between studies. Additionally, there is a lack of knowledge regarding the ecological consequences of BDM degradation products (Lambert and Wagner, 2017). Repeated tilling of BDM fragments into soil may alter the soil physical environment and act as a new source of carbon for microbes. In this regard, effects of BDMs on soils are unique compared to other plastics. The dearth of research directly comparing BDMs to PE renders it difficult to tease apart whether BDMs have an impact on soil microbes and their activities above and beyond what would be expected from a PE plastic film. The few available comparative studies show that microbial activity is enhanced under BDMs. This suggests that despite the fact that total carbon input from BDMs is minuscule, a stimulatory effect on microbial activity may contribute to soil microbial biomass and ultimately soil organic matter.

Several key gaps remain in our understanding of BDMs and their impacts on soil ecosystems. First, studies to date have focused on short term effects, generally one or two growing seasons, or acute toxicity, so long term effects are unknown. Second, the relationship between plastic composition and microbial responses needs exploration: different types of biodegradable plastics will likely differentially affect soil microbes, based on both the parent polymer composition and breakdown products. Third, additives have been demonstrated to leach out of plastic and affect plants; but their effects on soil microbes are unknown. Fourth, several studies have indicated that BDMs may stimulate decomposition; however, effects on nutrient biogeochemistry are largely unexplored. To address these knowledge gaps, long term studies are needed to assess soil health and sustainability impacts, particularly with respect to soil carbon and/or chronic toxicity effects. In addition, studies should include a direct comparison of PE to BDMs to determine whether BDMs affect soils differently than conventional plastic mulches. Addressing these knowledge gaps will provide much-needed information to growers and regulators on the safety and sustainability of BDMs for agroecosystems.

## Author Contributions

SB and JD conceived of the review topic and were responsible for final editing. SB, LM-C, AP, and JD all wrote portions of this review.

## Conflict of Interest Statement

The authors declare that the research was conducted in the absence of any commercial or financial relationships that could be construed as a potential conflict of interest.
